# The contribution of ethnic groups to Malaysian scientific output, 1982–2014, and the effects of the new economic policy

**DOI:** 10.1007/s11192-016-2139-3

**Published:** 2016-10-01

**Authors:** Grant Lewison, Sameer Kumar, Chan-Yuan Wong, Philip Roe, Richard Webber

**Affiliations:** 1 Department of Cancer Studies, King’s College London, Guy’s Hospital, Great Maze Pond, London, SE1 6RT UK; 2 Asia-Europe Institute, University of Malaya, 50603 Kuala Lumpur, Malaysia; 3 University of Malaya, 50603 Kuala Lumpur, Malaysia; 4 Evaluametrics Ltd, 157 Verulam Road, St Albans, AL3 4DW UK; 5 Department of Geography, King’s College London, Strand, London, WC2R 2LS UK

**Keywords:** Ethnicity, Onomastics, Economic policy, Malaysia, Scientific output

## Abstract

Malaysia has three main ethnic communities: Chinese, Indians and Malays. At independence in 1957, the Chinese dominated commercial life, and this led to ethnic tensions and finally riots. As a result in 1969 Malaysia introduced a “New Economic Policy” (NEP) to promote Malays in all areas of activity, and in particular to assist them to obtain basic and higher education. We examined the scientific outputs from Malaysia between 1982 and 2014 and classified the names of Malaysian researchers into one of these three groups and two others. There was a major increase in Malay participation in research, which has risen from 20 % of researchers in 1982–1984 to 65 % in 2012–2014, with corresponding declines in the percentages of Chinese and Indian authors, although their absolute numbers have increased because Malaysian scientific output has increased so rapidly in the last 10 years. The huge increase in Malay researchers contrasts with their presence in the Malaysian population which has remained stable at about 50 % since 1969.

## Introduction

### Malaysia: the country

**Fig. 1:**
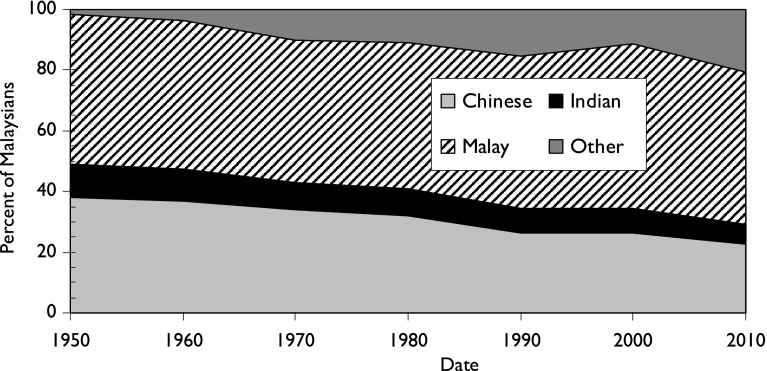
Estimates of the ethnic composition of the population of Malaysia from 1950 to 2010

Malaysia is notable for the multi-ethnic character of the country, where Malays (the largest group within Bumiputras, the name of the indigenous population) are living alongside two other communities, ethnic Chinese and ethnic Indians, who in 2010 comprised respectively 23 and 7 % of the population of 28 million. The Chinese comprised 38 % of the population in 1957, but since then their fertility rate, together with that of the Indians, has declined faster than that of the Malays, so their share of the population has gone down, see Fig. 1. However, as the population of Malaysia has expanded very rapidly, from 6.4 million in 1957 to approximately 31 million today (2016), all the communities have increased in size, including that of “others”, who include smaller Bumiputra populations and some recent immigrants from Bangladesh, Indonesia, the Philippines and Thailand.

The Federation of Malaya gained independence from Britain in 1957, and in 1963 Singapore and North Borneo joined and it became Malaysia. However in 1965 Singapore was separated from Malaysia and became an independent sovereign state. Tensions between the Malay and Chinese communities in 1969 led to serious riots. These were considered to be fuelled by the polarization of society along socioeconomic and, in particular, ethnic lines (Faaland et al. 2003). The Malaysian government thereafter developed the New Economic policy (NEP) in 1971 for a period of 20 years, with the prime aim of reducing poverty and achieving economic parity among the various ethnic communities (von Braun and Thorat 2014). It was succeeded by the National Development Policy (NDP). For the last four and a half decades, the NEP and NDP have shaped Malaysia’s socioeconomic development and political landscape (Gomez and Saravanamuttu 2013). The primary goal of NEP was national unity in a nation with many religious and ethnic groups.

Under the NEP, two major strategies were adopted (EPU 2016):

- To reduce and eradicate absolute poverty irrespective of race through raising income levels and increasing employment opportunities for all Malaysians; and
- To restructure society to correct economic imbalances so as to reduce and eventually eliminate the identification of race with economic function.

A major component of this policy was affirmative action (or positive discrimination) in favour of Bumiputras in both the private and government sectors (Jomo and Sundaram 2004). This included a significant push for higher education for Malays and preferential allocation of jobs (von Braun and Thorat 2014). The affirmative action in the education sector included the opening of higher learning institutions and scholarships for Bumiputra students (Malays), and a preferential quota system for admission in universities (Lee et al. 2013). Licences and other facilities for setting up industries were provided to the Malay community in order to increase Malay ownership of private enterprises (von Braun and Thorat 2014). Thus, in practice, the NEP policies were seen as pro-Malay, the largest indigenous ethnic community (Jomo and Sundaram 2004).

Undoubtedly, these initiatives have had a positive effect. As a matter of fact, poverty, unemployment and infant mortality have dropped significantly over the years (Gomez and Saravanamuttu 2013). Yet these improvements have not been without their share of persistent criticisms that the NEP and its successive plans (i.e. the National Development Policy and the National Vision Policy) have also created intra-ethnic income disparities among Bumiputras and a serious brain drain (Gomez and Saravanamuttu 2013; Jomo and Sundaram 2004; von Braun and Thorat 2014).

**Fig. 2:**
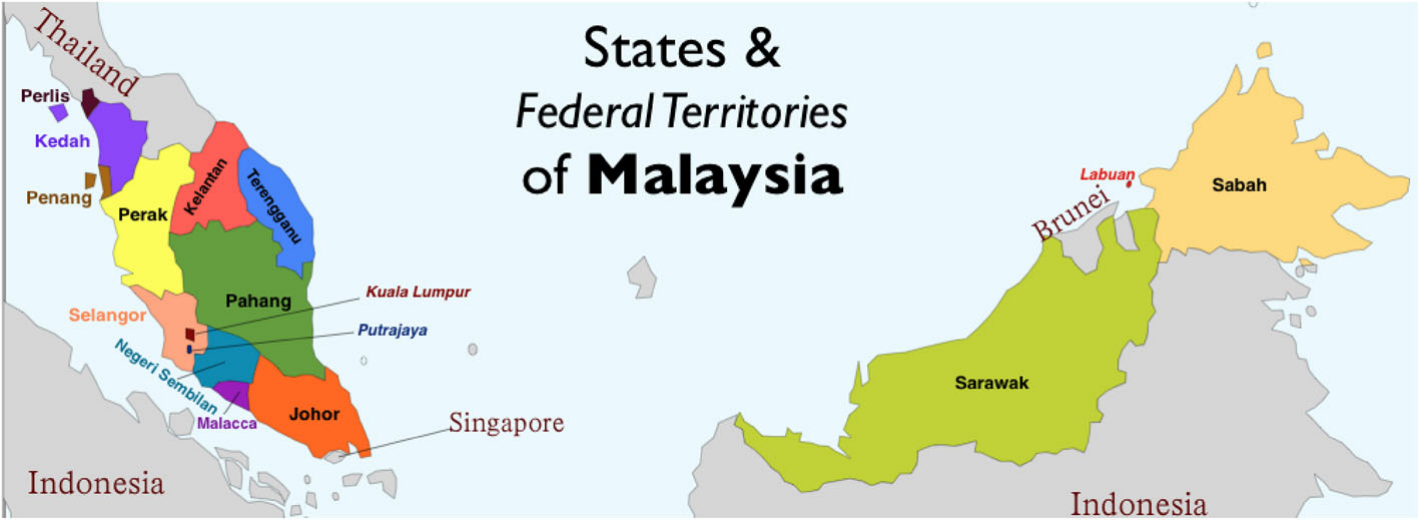
The 11 states and 3 federal territories of Malaysia

We wondered if these policies had been effective in helping the Malays to participate more in research and to publish more papers in international journals over the last four decades. Another subject for our enquiry was to see if the ethnic balance among researchers varied with geography—Malaysia has 13 states, of which two are within the island of Borneo and the other 11 in the peninsula (see map, Fig. 2), and three federal territories including the capital and a small island off the coast of Borneo.

Malaysia is a country that is changing rapidly. Its population has doubled in 30 years and it now enjoys an average income *per caput* of $10,073 (IMF data 2015), just above the world average and ahead of Mexico and Turkey. Its economy, which used to be based on tin, rubber, palm oil and other agricultural products, is now diversified and agriculture only accounts for 7 % of the total (with industry contributing 37 % and services 56 %).

## Science in Malaysia

**Fig. 3:**
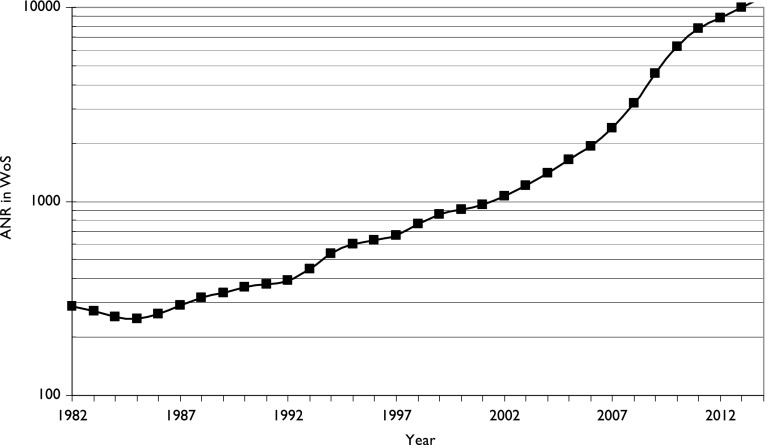
The growth in output of scientific research in Malaysia (articles, notes and reviews in the Web of Science: SCI-E, SSCI and AHCI). Three-year running means, log scale for ordinate

Malaysia has also greatly increased its commitment to scientific research, and Fig. 3 shows the growth in its output between 1982 and 2014 (articles, notes and reviews in the Web of Science, WoS). Output stagnated in the early 1980s, but then increased at about 10 % per year until 2005, when it surged ahead at an annual growth rate of 24 %. Under the Wawasan 2020 program, Malaysia aspires to be a developed nation by the year 2020 (Kumar and Jan 2013). Research and development is an important aspect of this programme to take the country towards this goal. The 9^th^ and 10^th^ Malaysia plans (9MP and 10MP) have allocated substantial funds for investment in research and development and for carrying out fundamental research in both hard and soft sciences. The five public research universities—Universiti Malaya, Universiti Sains Malaysia, Universiti Putra Malaysia, Universiti Kebangsaan Malaysia and Universiti Teknologi Malaysia—have been major beneficiaries of these funds. Consequently, these universities have also managed to produce the bulk of research papers for the country.

There have been a number of studies of Malaysian scientific output. Some have examined domestic production (Bakri and Willett 2011; Salmah 2015); others have compared Malaysian research output with that of other countries or regions (Nguyen and Pham 2011; Sarwar and Hassan 2015). A few studies have looked at research collaborations at the micro, meso and macro levels (Kumar and Jan 2013), or at the socio-academic parameters that are influential in bringing researchers together (Kumar and Jan 2015).

Nguyen and Pham (2011) found that Malaysia accounted for 16 % of the total papers produced by ASEAN nations and, along with Thailand, registered the highest rate of growth in research output. Malaysian authorship trends follow a typical power law, where a large number of authors produce a single paper while a few produce many. For example, nearly 64 % of researchers in biomedical research produced just one paper and 16 % produced 30 or more papers (Zainal and Zainab 2011). An investigation last year (Sarwar and Hassan 2015) showed that the research production of Malaysia in S&T areas was third in the Islamic world, after Iran and Turkey. The study noted Malaysia’s impressive research output in the energy sector, probably because of its dependence on this sector.

Several studies have looked at the research output of individual fields, such as engineering (Salmah 2015), computer science (Bakri and Willett 2011), toxicology (Zyoud et al. 2014), palm oil (Abrizah et al. 2012) and library & information science (Yazit and Zainab 2007). The majority of these studies found a significant increase in the output of papers in their respective fields over the years. For example (Salmah 2015) analysed Scopus data comprising 11,819 bibliographic records in the field of engineering and found that Malaysian researchers had improved their research output considerably since 2007 with a consistent trend towards increased collaboration. Similarly, in toxicology research, the number of papers increased tenfold from 2003 to 2012 (Zyoud et al. 2014).

A few studies have looked at research outputs from the perspective of collaborations and networks. Kumar and Jan (2013) carried out a study of research collaborations in the business and management field in Malaysia over the three decades from 1980 to 2010 and found, *inter alia*, that the authors had become twice as collaborative in 2001–2010 compared to 1980–1990.

Although co-operation on education between its member states remains an important objective of ASEAN, of which Malaysia is a prominent member, it collaborated relatively little with its ASEAN neighbours compared with non-ASEAN countries. Kumar and Jan (2014) used another network perspective to examine collaborations and compared the collaborative patterns of Malaysia-based authors in the field of energy and fuels with those of another OIC country—Turkey. Their study found that the centrality measures had significant correlation with research production. However, the results were found to correlate far more with the Malaysia network than with those of the Turkey network.

Malaysian authors rely on established forms of scholarly communication, but are also quick to exploit new channels such as social media or new journal models that have not yet found their way into the mainstream scholarly community (Abrizah et al. 2015). In another study (Shin et al. 2014) it was discovered that Malaysian researchers returning home after obtaining advanced degrees abroad were likely to be less productive in hard sciences than those researchers who stayed at home.

Malaysia is a multi-ethnic nation, where people of three major ethnicities (Malay, Chinese and Indians) live and work alongside one another. However, studies on Malaysia’s research productivity based on ethnicity or race are few. (It is worth mentioning here that ethnicity is a form of identity depending on where a person’s ancestors came from. Nowadays, race and ethnicity are often used interchangeably, although in the strict sense of the term, race primarily refers to physical traits and ethnicity refers to cultural traits.). Among these few ethnicity-based studies on Malaysia is a small study looking at the ethnic balance of Malaysian researchers in a research-intensive university, based on socio-academic patterns, such as race, professional position, gender, etc. This study did not find any significant preference for researchers to associate with other researchers of the same race.

## Scientific personnel: ethnicity and sex

There has been an increasing interest in the composition of the scientific labour force in many countries. In part this is to try and improve the representation of women, who now comprise close to half, or even more, of undergraduates studying science, but whose proportion steadily declines with seniority. The other aspect of study is the ethnic composition of the scientists in a country, or their national origin. This is important as it can show how open and welcoming a country is to researchers from abroad, which may well stimulate the production of high quality science. For countries where there are several distinct ethnic communities, such studies can also show if progress has been made in the improvement of the opportunities for members of groups previously subject to discrimination such as non-whites in South Africa (Lewison and Jacobs 2011). These studies can also reveal whether occasional episodes of “ethnic cleansing” have affected the scientific community (Lewison and Igic 1999), and what contribution immigrants from particular world regions are making to the scientific output of their new host countries (Basu et al. 2012). So it seemed appropriate to employ this approach to Malaysia.

Some such studies are based on the characterisation of research personnel by their sex from databases held by central government, for example in Brazil (Leta 2003; Batista and Leta 2009), Italy (Abramo et al. 2009), Russia (Markusova 1997) and Spain (Mauleon et al. 2008), which may give their sex directly. However, most such studies depend on peoples’ names (Hopkins et al. 2013). Surname as a proxy was used to show the ethnicity of authors of publications and inventors of patents in biomedical research (Kissin 2011; Kissin and Bradley 2013). The differences in citations to papers by men and women in Iceland (Lewison 2001), Poland (Webster 2001), South Africa (Prozesky and Boshoff 2012), and Russia (Lewison and Markusova 2011) were also examined by this method. A major study covering all countries and all fields of science was published in *Nature* (Larivière et al. 2013). More recently, a study of lung cancer researchers world-wide used surnames and given names to show the variation in the percentage of women in different countries, and the contribution of immigrants to their research output (Lewison et al. 2016).

## Methodology

The primary source of data for this study was the Web of Science (WoS) published by Thomson Reuters. We sought and downloaded to file the bibliographic details of articles, notes and reviews with at least one address in Malaysia from four three-year periods: 1982–1984; 1992–1994; 2002–2004; and 2012–2014. Papers were taken from three indexes: the Science Citation Index Expanded, the Social Sciences Citation Index, and the Arts and Humanities Citation Index. Since 2007, the WoS has tagged the names of the authors of papers with their individual addresses, so that authors affiliated with institutions in a specified country can be identified. This means that for the last triennium, it was possible to identify the Malaysian authors on multi-national papers, but this could not be done for the first three sets of papers, which in order to exclude any foreign authors we limited to ones with no international collaboration.

The bibliographic data from the papers were converted from text files to an Excel file by means of a special program, developed by PR. This had a feature that identified the authors from a specified country (here, Malaysia) on papers where the authors were tagged with their addresses. However, it could not cope with some physics papers with more than 1000 authors, which have appeared during the last few years, notably from CERN in Geneva, so these papers had to be removed (the Malaysian presence in them would have been very small).

The remaining papers were characterised by their major fields, based on a scheme originally developed by CHI Research Inc. There are 14 of these, listed in Table 1; all are based on the journals in which papers are published and there are no overlaps (as with the WoS categories). The cities in the Malaysian addresses were also coded by the state or federal territory in which they were located, see Table 2. Many of them were state capitals, but there were numerous small towns as well.

**Table 1:** List of major fields among which WoS papers are distributed

Biology	Engineering and techn	Physics
Biomedical research	Health sciences	Professional fields
Chemistry	Humanities	Psychology
Clinical medicine	Mathematics	Social sciences
Earth and space	Multidisciplinary

**Table 2:** List of Malaysian states and federal territories with their official trigraph codes

State/territory	Code
Johor	JHR
Kedah	KDH
Kelantan	KTN
Kuala Lumpur	KUL
Labuan	LBN
Malacca	MLK
Negeri Sembilan	NSN
Pahang	PHG
Penang	PNG
Perak	PRK
Perlis	PLS
Putrajaya	PJY
Sabah	SBH
Sarawak	SWK
Selangor	SGR
Terengganu	TRG

The names of all the Malaysian authors were coded into one of four categories based on a very large database of family names maintained by RW comprising nearly four million names. For the 2012–2014 papers, given names were also available from the WoS and this allowed them to be compared with a large thesaurus of 0.7 million personal names. The Chinese category (CHI) included both Cantonese and Mandarin names, and also the short names from Korea, Singapore and Vietnam. The Indian category (IND) included names from 13 different states or regions, plus Sri Lankan names (the majority of the Malaysian Indian population are Tamils some of whom would have come from Ceylon), plus some other non-Indian Hindu names. The Malay category (MAL) included ones characteristic of Malaysia plus all Muslim names, including ones from Iran and Turkey. Some names could be categorised as European (EUR): they include some Bumiputras in the Borneo states who are Christian and adopted European names. All other names were classed as Other (OTH). There are a number of foreign researchers living in or visiting Malaysia, but we have assumed that they represent only a small fraction of the Malaysians.

Each name in the lists of authors was then characterised as CHI, EUR, IND, MAL or OTH, and the fractions from each group on each paper were calculated. This enabled us to determine the fractional counts of contributions from each of the five groups to any given set of papers, such as those from a triennium, from a state or federal territory, or from a major field. However, some states or territories had very few papers, and some major fields were also poorly represented.

The results are presented separately for the first three triennia (1982–1984, 1992–1994 and 2002–2004) and for the last one, as the papers in the latter triennium included ones with international collaboration, and this was also analysed in order to see if the different groups had different foreign partners.

## Results: from 1982 to 2004

In these three three-year periods, there were a total of 5802 papers, of which 3364 (58 %) had no international co-authorship and were retained for onomastic analysis. Table 3 shows the numbers of researchers from the five groups in each triennium, together with their contributions on a fractional count basis for each paper.

**Table 3:** Numbers of researchers in Malaysia from five ethnic groups, 1982–1984, 1992–1994 and 2002–2004, and their total fractional contributions: all fields of science

Period	Researcher names						Contributions (Fractional count)					
	CHI	EUR	IND	MAL	OTH	ALL	CHI	EUR	IND	MAL	OTH	ALL
Numbers												
82–84	263	148	108	138	35	692	237.9	159.6	84.7	98.2	31.6	612
92–94	356	155	181	458	136	1286	299.9	90.8	115.2	282.8	75.3	864
02–04	812	326	293	1277	316	3024	527.3	166.4	180.1	868.5	145.7	1888
Percent												
82–84	38.0	21.4	15.6	19.9	5.1		38.9	26.1	13.8	16.0	5.2	
92–94	27.7	12.1	14.1	35.6	10.6		34.7	10.5	13.3	32.7	8.7	
02–04	26.9	10.8	9.7	42.2	10.4		27.9	8.8	9.5	46.0	7.7	

The contributions run approximately in parallel with the numbers of researchers from the five groups. The Chinese relative contribution has declined by rather more than a quarter, and that of the Europeans by nearly two-thirds, although their actual numbers have increased substantially. The Indians have also seen a decline in their relative contribution. However the Malays have clearly done spectacularly well, with a nine-fold increase in numbers of researchers, and their relative contribution almost tripled between 1982–1984 and 2002–2004.

The next analysis was in terms of the major fields, see Table 2. It turned out that the different groups had quite different interests, so that Malays were strong in engineering and technology, and to a lesser extent in physics and earth and space, but relatively weak in clinical medicine. The Chinese were strong in biomedical research and chemistry, but less so in engineering and technology and earth and space. The Indians excelled in clinical medicine but not in chemistry; this contrasts with the Indian national strength in this field. The data are in Table 4.

**Table 4:** Numbers of researchers in Malaysia in different major fields of science, and their fractional contributions; 1982–1984, 1992–1994 and 2002–2004 combined

	Papers, FRAC CT						Relative commitment				
Field	CHI	EUR	IND	MAL	OTH	ALL	CHI	EUR	IND	MAL	OTH
Clinical medicine	218	82	115	197	60.1	672	1.03	0.98	*1.51*	0.79	1.19
Biology	180	102	50.3	190	55.6	578	0.98	*1.42*	0.77	0.89	1.28
Chemistry	216	61.4	40.8	224	29.1	571	1.19	0.87	**0.63**	1.06	**0.68**
Engineer and Tech	120	46.4	59.5	270	42.7	539	**0.70**	**0.69**	0.98	1.35	1.06
Biomed research	115	25.7	36.6	102	20	299	1.22	**0.69**	1.08	0.92	0.89
Physics	69.5	15.1	20.5	90.5	9.3	205	1.07	**0.59**	0.89	1.19	**0.60**
Earth and space	33.6	25.2	11.5	63	8.7	142	0.75	*1.43*	0.72	1.19	0.82
Others	113	59.6	46.3	113	26.9	358	0.99	1.34	1.14	0.85	1.00
Total	1065	417	380	1249	252	3364					

Relative commitment cells with values >1.41 shown in bold; those with values <0.71 in italics

Table 5 shows the composition of the research workforce in the major states and federal territories over the whole period of analysis. The distribution by state/federal territory is very skewed, with almost three quarters of all papers coming from Kuala Lumpur and Selangor, which surrounds the capital (see Fig. 2). There are also pronounced differences in the ethnic composition of the different states. The Chinese presence is greatest in Malacca, Kuala Lumpur (the capital), Penang and Sarawak. The Indians are most visible in Malacca and Kelantan, and the Malays in Johor and Selangor, the latter being the most populous state. The Europeans (who include some indigenous populations in the island of Borneo, v.s.*)* are most prominent in Sabah and Sarawak. It is rather surprising that these percentages do not agree with the distribution of the main ethnic groups according to the 2000 census. For the Chinese, there is a moderately positive correlation (*r* ^2^ = 0.46) but for the Indians and Malays the correlation is actually negative.

**Table 5:** Outputs of eight leading Malaysian states and federal territories in the WoS, 1982–1984, 1992–1994 and 2002–2004, with fractionated counts of contributions to these papers by five ethnic groups, and their percentage presence in each state or federal territory

				Contributions to papers					Relative presence				
Code	INT	FRAC	% MY	CHI	EUR	IND	MAL	OTH	CHI	EUR	IND	MAL	OTH
KUL	1548	1424.1	42.3	549	194	184	397	100	39	14	13	28	7.0
SGR	1215	1066.5	31.7	253	107	73.7	544	88.7	24	10	6.9	51	8.3
PNG	492	455.4	13.5	169	49.3	50.1	163	24.1	37	11	11	36	5.3
KTN	104	99.6	3.0	5.89	13.3	25.9	38.9	15.6	5.9	13	26	39	16
JHR	98	80.5	2.4	14.5	8.73	4.65	47.3	5.33	18	11	5.8	59	6.6
MLK	82	66.8	2.0	30.7	9.92	20.2	5.75	0.33	46	15	30	8.6	0.5
SBH	60	49.2	1.5	11.0	12.6	5.95	13.5	6.14	22	26	12	27	12
SWK	57	42.9	1.3	15.7	9.65	3.32	9.45	4.87	37	22	7.7	22	11

For state codes, see Table 2

## Results: 2012–2014 Malaysian papers

**Fig. 4:**
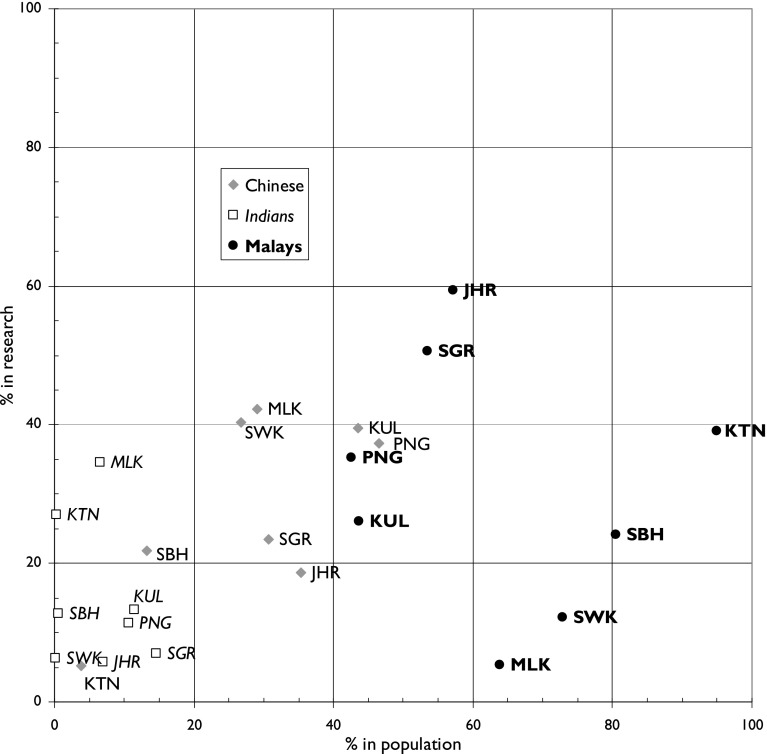
Comparison between presence of Chinese, Indians and Bumaputras or Malays in the population of eight Malaysian states (including the capital) and their research activity. *For state codes, see Table* 2

As Fig. 3 indicates, there has been an enormous expansion of Malaysian scientific output recently, and in the 3 years 2012–2014 there were 29,714 papers in the WoS, of which 15,254 (51 %) were from Malaysia only without international collaboration. (There were 20 papers apparently from Malaysia, but their addresses were actually from other countries, and they were mistakenly attributed to Malaysia by the WoS.) The analysis below is of all the papers as the Malaysian authors can be separated from international ones. The leading partners for Malaysian researchers were the UK (1968 papers, 6.6 %), Australia (AU, 1774, 6.0 %), Iran (IR, 1736, 5.8 %) and the USA (US, 1536, 5.2 %). There were also collaborations with other Islamic countries: notably with Saudi Arabia (SA, 1020, 3.4 %) and Pakistan (PK, 687, 2.3 %). However there was rather less than expected with China (CN, 888, 3.0 %) and with India (IN, 1321, 4.5 %) (Fig. 4).

**Fig. 5:**
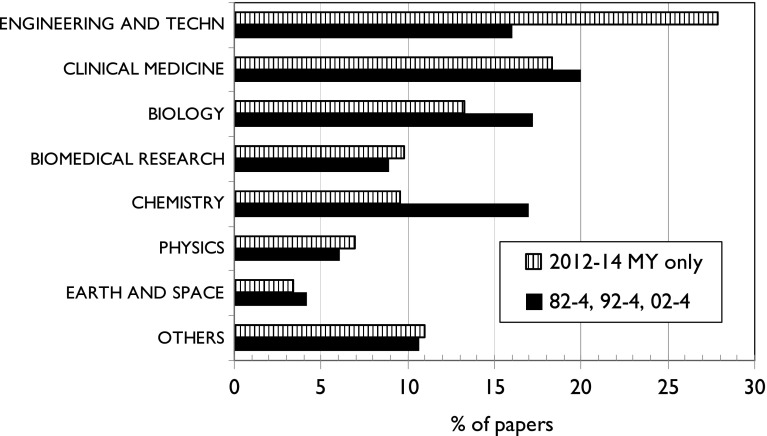
Major fields of Malaysia-only papers from 2012 to 14 compared with three earlier triennia

There has also been a noticeable change in the major fields of the papers. Figure 5 compares the balance between them for the first three triennia and the last one—the data for the latter pertaining only to the 15,254 domestic papers. The major change has been the big rise in engineering and technology and the decline in chemistry and to a lesser extent in biology. This will have favoured the Malays and disadvantaged the Chinese and Europeans, but may have simply reflected the changes in the population of the country and of scientists.

**Fig. 6:**
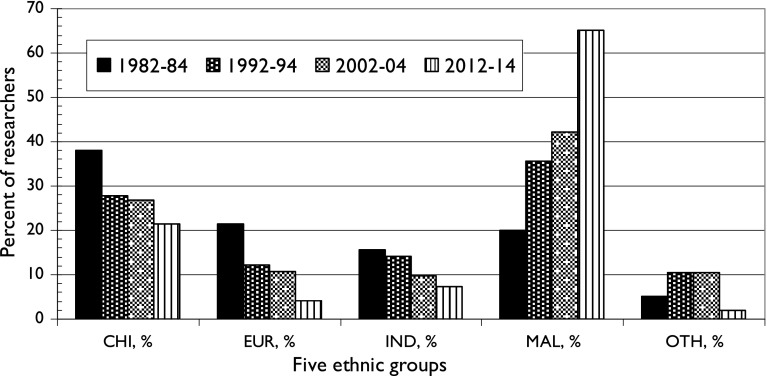
Ethnic composition of Malaysian research workforce, 1982–2014

The analysis of names revealed that, as expected, there were relatively more Malays than in earlier years and fewer of the other groups, see Fig. 6. The Malays or Bumiputras are now dominant, with almost two-thirds of researchers being Muslim according to their names, and Europeans and “others” each account for less than 5 % of the total. Although the percentages of Chinese and Indians have continued to decrease, in absolute terms their numbers have actually gone up, and there were 8163 Chinese names and 2740 Indian names on 2012–2014 papers. (The actual numbers of researchers will have been less as some researchers gave only their initials on some papers but given names on others and so will have been counted twice.)

The contributions of the five groups to Malaysian scientific output are shown in Table 6. They are shown both as integer counts and as fractional ones. It appears that the Indians are the most productive, with 2.92 papers per name, and the Europeans and “others” less so, with 2.63 and 2.42 papers per name. (No figures are given for foreign authors on an integer count basis.) The fractional counts were obtained by means of a macro written by PR that assigned a fractional count to each paper of Malaysian author names from each of the five groups, and foreign authors. Some Malaysian authors wrote papers from two addresses, one in Malaysia and one abroad. For purposes of analysis they were treated as being divided into two, and both their Malaysian and foreign contributions were halved.

**Table 6:** Contributions to Malaysian research in 2012–2014 from each of the five ethnic groups and from foreign researchers, integer counts (INT) and fractional counts (FRAC)

Group	N authors	% of MY authors	INT count	Mean	FRAC count	% of total	Mean
CHI	8163	21.6	21,745	2.66	3971	13.4	0.49
EUR	1535	4.1	4036	2.63	1365	4.6	0.89
IND	2740	7.2	7991	2.92	1562	5.3	0.57
MAL	24,641	65.1	67,753	2.75	13,916	46.8	0.56
OTH	751	2.0	1820	2.42	1113	3.7	1.48
Foreign					7787	26.2	
Total	37,830		103,345	2.73	29,714		

**Fig. 7:**
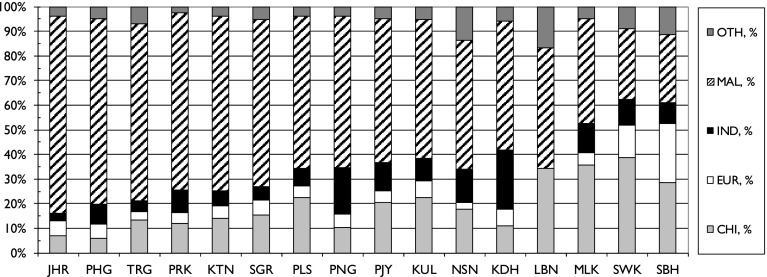
The fractional contributions by the five ethnic groups to research outputs from the 16 Malaysian states and federal territories, 2012–2014. *For codes, see Table* 2 *. States are ranked by percentage of Malay presence*

**Fig. 8:**
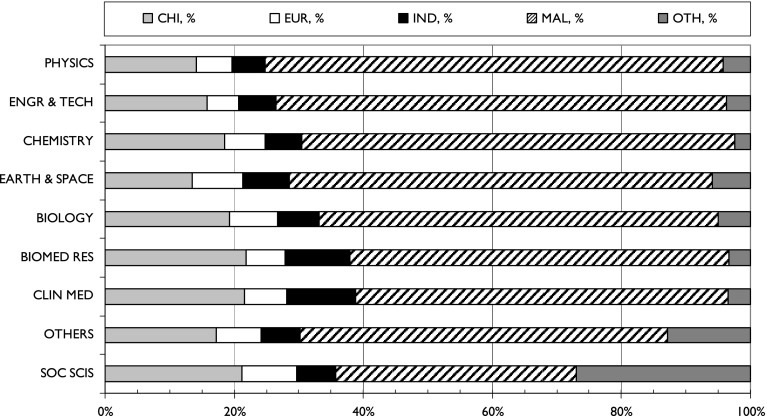
The contributions of the five ethnic groups to Malaysian science in different major fields, 2012–2014. *Major fields are ranked by percentage of Malay presence*

**Fig. 9:**
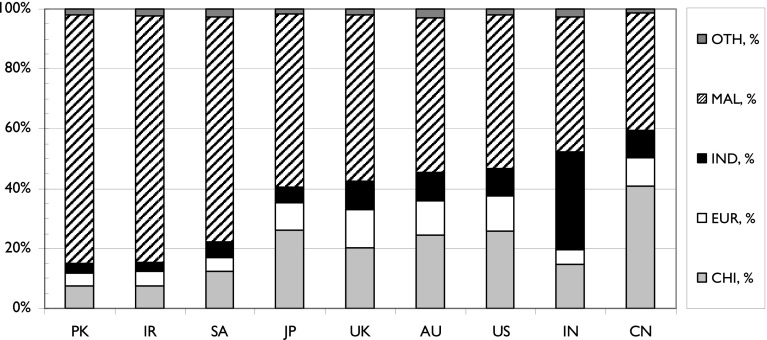
The contributions of the five ethnic groups to Malaysian science with its major foreign partners, 2012–2014. *Foreign partners are ranked by percentage of Malay presence. PK* Pakistan, *IR* Iran, *SA* Saudi Arabia, *JP* Japan, *AU* Australia, *US* United States of America, *IN* India*, CN* China

This attribution of ethnic groups (and foreign authors) to each paper allowed the analysis of these groups’ contributions to each Malaysian state, to each major field, and to papers co-authored internationally with the leading partner countries (see above). The results are shown in Figs. 7, 8and 9.

The Malay contribution to research has clearly expanded rapidly in many states, as a comparison of Table 5 and Fig. 7 shows, but not in all. For example, it rose from 28 % in Kuala Lumpur (KUL) in 1982–2004 to 56.5 % in 2012-14, and in Malacca (MLK) from 8.6 to 42.8 %, but in Sarawak (SWK) from 22 to 28.5 % and in Sabah (SBH) only from 27.4 to 27.5 %. So it appears that the increased Malay participation in research was much greater in the Peninsula than in the two Borneo states. The Chinese contribution has declined as a percentage of the total output in KUL from 39 to 22 %, but in SWK it has increased slightly from 36.5 to 39 %. The Indian contribution has decreased in most states, but in Penang (PNG) it went up from 11 to 19 %.

With regard to the major fields of Malaysian research, Fig. 5 shows that engineering and technology has expanded the most, and this is a field in which Malays have traditionally been strong (Table 4). In 2012–2014, they published almost 70 % of the total output of 6132 papers, whereas in 1982–2004, their contribution was only 50 %. In physics, another area of strength, their contribution went up from 44 to 71 %. There has been a doubling of Malay representation in clinical medicine, a field traditionally dominated by the Indians, from 29 to 58 % in 2012–2014. It is still the field in which the Indians in Malaysia make the largest of their contributions, together with biomedical research, but they are now only minor players compared with the Malays.

Finally, Fig. 9 shows the relative contributions of the five ethnic groups to Malaysian research carried out in collaboration with other countries. It is striking but not surprising that the Chinese dominate research done with researchers from China (41 % of the papers), that the Indians favour collaboration with India (33 %) and the Malays coöperate most readily with three other Muslim nations, Pakistan (83 %), Iran (82 %) and Saudi Arabia (75 %). The small number of European researchers favour the UK (13 % of the total), Australia and the USA (12 % each).

## Discussion

This study has demonstrated that the Malays within Malaysia, whose numbers have expanded greatly since the early 1980s, are now dominating scientific research, and that their situation has changed greatly over the last 40 years. This is graphically illustrated in Fig. 6, where their contribution has risen from a minority (20 %) in 1982–1984 to their rather dominant position in 2012–2014 when they were responsible for 65 % of the (much larger) Malaysian total number of publications. However, their situation has improved much more in the Malaysian peninsula than in Borneo. It appears that their preference for engineering has meant that Malaysian progress in science has been uneven, with the biological sciences and chemistry suffering the biggest declines.

The methodology served to categorise the Malaysian researchers on the basis of their names, but the initial allocation of broad ethnic categories turned out not to be as accurate as expected. Individual inspection (by GL) of the categories of the 37,830 names of Malaysian researchers in 2012–2014 (some of them were clearly the same person, sometimes with initials and sometimes with their given names) showed that there were many anomalies, and this is illustrated in Table 7. The biggest corrections were to the European and Chinese names, as originally allocated, some of which appeared to be of Muslim origin and were re-classified as Malay.

**Table 7:** Numbers of changes made to initial allocation of Malaysian researcher names to ethnic categories based on individual inspection

Group	Original	Revised	Change	New CHI	New EUR	New IND	New MAL	New OTH
CHI	9193	8163	−1030	7574	21	15	1483	100
EUR	2883	1535	−1348	495	1426	71	787	104
IND	3102	2740	−362	19	14	2586	430	53
MAL	22,194	24,641	2447	49	58	55	21,763	269
OTH	458	751	293	26	16	13	178	225
Total	37,830	37,830	0	8163	1535	2740	24,641	751

We did notice that the tagging of authors with their addresses by the WoS was by no means perfect, and as a result the numbers of individual authors so tagged in the 2012–2014 papers did not always equal the numbers of authors in the Authors column of the spreadsheet. In this analysis, we used the column of data containing the authors tagged with their addresses, so that we could perform an analysis of the individual Malaysian states. This meant that the fractionation of the papers by country differed from the traditional one based on numbers of addresses because in this study it was based on numbers of authors from the different states (and countries). The methodology developed for this study can be modified and used for other bibliometric analyses, so that a country’s (or an institution’s) contribution to a research paper can be based on the number of authors rather than the numbers of addresses, which is in principle more appropriate. It is doubtful if this will make a significant difference to the allocation of credit to individual countries or institutions except when there are very small numbers of papers, but the matter should be investigated and will be the subject of a future communication.

## Conclusion

The main conclusion is that Malays have greatly increased their participation in research in Malaysia over the last 32 years, from 20 % in 1982–1984 to 65 % in 2012–2014. However this increased participation was much greater on the Peninsula than in the two Borneo states. One consequence is that the balance of Malaysian science has shifted towards engineering and technology, in which the Malays are strong, from chemistry. The pattern of collaboration with foreign countries still strongly reflects the ethnic divisions within Malaysia, with the Malays favouring other Muslim countries, the Indians India and the Chinese, China.
